# Evaluating the Use of Google Street View to Visually Verify the Locations of Cannabis Retailers in the United States Extracted from Websites, 2015–2018

**DOI:** 10.1007/s12061-026-09823-1

**Published:** 2026-03-28

**Authors:** Danielle Haley, Magdalena Pankowska, Michael Williams, Hannah Cooper, Andrew Edmonds, Kyle Nameth, Matthew Mahlan, Yen-Tyng Chen

**Affiliations:** 1 School of Public Health, Boston University, Boston, United States; 2 Northeastern University, Boston, United States; 3 Emory University, Atlanta, United States; 4 University of North Carolina at Chapel Hill, Chapel Hill, United States; 5 Rutgers, The State University of New Jersey, New Brunswick, United States

**Keywords:** Cannabis, Cannabis legislation, Cannabis retail, Google street view

## Abstract

Our ability to advance public health and policy responses to cannabis legalization is limited by a lack of geographic data on cannabis retailers across states and over time. This study evaluated the feasibility and utility of using Google Street View (GSV) to: 1) visually verify cannabis retailers locations extracted from websites and (2) create a historical retailer list. We extracted and deduplicated cannabis retailer addresses from 6 websites advertising medical and adult use cannabis retailers across 13 US states in February 2019. We visually verified the locations of cannabis retailers in 2015, 2016, 2017 and 2018, summarizing results by state and year. We assessed proportions of verified retailers within and across the legislative period in which states first legalized medical cannabis (ballot [1996–1999], early [2000–2008], late [2009–2018]) using a Chi-square test. Of 1,739 retailers, 74% were visually verified at least one year between 2015 and 2018. Verification was ≥ 74% in all but California and Florida in 2018. Visual verification prior to 2017 was low, largely due to missing GSV images. Proportions of visually verified retailers varied between states legalizing medical cannabis in the ballot (*p* < 0.001), but not in the early (*p* = 0.236) and late legislative periods (*p* = 0.585). GSV virtual audit methods may be appropriate for visually verifying cannabis retailers contemporaneously, but are not appropriate for reconstructing historic retailer lists. Some states (e.g., California) may require the use of additional verification methods. Future studies should assess the feasibility of using GSV in conjunction with novel methods for processing data (e.g., machine learning).

## Background

### Context: Cannabis Legalization in the United States

In 2024, most United States (US) jurisdictions had legalized the sale of cannabis for medical or adult/recreational use (National Academies of Sciences et al. 2024). Regulated cannabis retail markets in the US have grown in response to widespread legalization and increasing prevalence of cannabis use (Berg et al., 2018; Freisthler et al., 2013; Pacula & Smart, 2017). The number of operating cannabis retailers has drastically increased in the past decade. For example, the number of cannabis retailers in Washington state, where medical and adult-use cannabis sales were legalized in 1998 and 2012, respectively, increased by 400% between 2014 and 2017(Amiri et al., 2019; Prescription Drug Abuse Policy System, 2024a, 2024b). Nationally, medical and adult use cannabis sales exceeded $30 billion in 2024, a 63% increase from 2020 sales (Whitney Economics, 2025).

Cannabis use may offer therapeutic benefits (e.g., pain management) while also posing harms (e.g., unintended injury, adverse mental health), warranting ongoing research and evaluation to inform clinical practice and policy responses (National Academies of Sciences et al. 2017, Bobitt et al., 2023; Livne et al., 2023, Haley et al. 2025). A growing body of research suggests greater physical proximity to cannabis retailers is positively associated with cannabis use and associated harms, though most of this research has been limited to a single year and state (Freisthler & Gruenewald, 2014; Freisthler et al., 2013; Pacula & Smart, 2017; Shi et al., 2016). The location of cannabis retailers in the US is shaped both by social (e.g., greater neighborhood poverty) and economic forces (e.g., greater neighborhood retail activity) (Freisthler & Gruenewald, 2014; Freisthler et al., 2013; Pacula & Smart, 2017; Shi et al., 2016). Similar social forces are thought to create localized risk environments promoting unhealthy substance use (Cooper et al., 2013; Dasgupta et al., 2018). Given the rapidly expanding and heterogenous regulatory approach to cannabis legalization, longitudinal studies across multiple states are needed to better understand the impacts of legalization on individuals and communities.

### Challenge: Measuring Cannabis Retail Environments

Lessons from tobacco and alcohol regulation highlight the importance of retail sales environments in subsequent use and harms, elevating the importance of understanding the role of cannabis retail markets on the health impacts of cannabis legalization (Berg et al., 2018, National Academies of Sciences et al. 2024). For example, limiting the density of alcohol outlets and setting minimum distance requirements from certain establishments (e.g., schools, churches), have emerged as evidence-based approaches to reducing alcohol-related harms (Stockwell et al., 2020). Although it is possible that similar approaches will provide effective in maximizing the benefits while minimizing harms of cannabis, our ability to advance public health and policy responses to cannabis legalization is limited by a lack of data. Cannabis science has confronted substantial challenges in measuring geographic access to retailers (Freisthler & Gruenewald, 2014; Shi et al., 2016). To our knowledge, comprehensive databases capturing the locations of cannabis retailers over time, which are needed for spatial analyses and other place-based analytic approaches (e.g., multilevel modeling), are not readily available. Because cannabis is federally prohibited, no federal agency is delegated with monitoring cannabis retailers systematically (e.g., creating a national database) (Cao et al., 2020; Pacula & Smart, 2017). State-level cannabis retailer licensing databases must be accessed through each individual state licensing agency, often requiring lengthy formal data acquisition requests. In addition, within states legalizing both medical and adult use sales, licensing may fall under separate agencies, amplifying data collection burden. Further, licensing data are collected for regulatory purposes and are updated infrequently, necessitating extensive data verification methods to identify operational cannabis retailers (Cao et al., 2020; Pedersen et al., 2018). As a result, collecting retailer data from multiple state licensing agencies may not be feasible or practical.

Creating retailer lists by extracting cannabis retailer names and locations from websites advertising medical or adult-use cannabis retailers, such as Leafly.com and Weedmaps.com, offers a more efficient way to collect cannabis retailer location data across multiple states (Cao et al., 2020; Pedersen et al., 2018, 2020; Shih et al., 2019; Unger et al., 2020). However, websites overestimate the actual number of cannabis retailers, necessitating additional data cleaning and validation procedures (Pedersen et al., 2018, 2020; Williams et al., 2023). Past studies verifying website-derived cannabis retailer lists in Los Angeles, California found only half of retailers initially extracted from websites could be verified through online procedures (e.g., reviewing retailer websites, conducting Google and Yelp searches) (Pedersen et al., 2018, 2020). Notably, most studies verifying retailer lists have largely been limited to single states or cities, allowing for more extensive data verification methods (e.g., site-visits, calling retailers, reviewing online sources) (Freisthler et al., 2016; Pedersen et al., 2018, 2020).

### Contribution to the Field: Evaluating the Utility and Feasibility of using Google Street View (GSV) Virtual Audit Methods to Systematically and Retrospectively Validate Cannabis Retailer Locations Extracted from Online Sources

Given the scale of cannabis legalization in the US, there is a need to explore whether less resource-intensive methods are suitable for collection and verification of cannabis retailer locations. One promising approach is to verify the physical locations of cannabis retailers extracted from websites using GSV virtual audit methods. GSV methods are a reliable, safe, cost-, and time-effective alternative to traditional in-person audit methods. GSV has been widely used to conduct virtual neighborhood “walk throughs” for characterizing neighborhood built environments, such as public spaces (e.g., parks, playgrounds), food environments (e.g., convenience stores, grocery stores, alcohol outlets), and general land use (e.g., high-rise housing, commercial/industrial units) (Chen et al., 2016; Clarke et al., 2010; Nesoff et al., 2020; Rundle et al., 2011; Rzotkiewicz et al., 2018). Rundle and colleagues compared field audit and GSV audit methods across seven neighborhood environment constructs (e.g., pedestrian safety, infrastructure for active travel, social and commercial activity); GSV demonstrated high concordance with field audit for physical environments (Rundle et al., 2011). GSV has been leveraged in past work examining the associations between retail environments (e.g., alcohol outlets, tobacco stores, pharmacies) and substance use (Chen et al., 2016; Clarke et al., 2010; Crawford et al., 2019; Pliakas et al., 2017; Rundle et al., 2011; Rzotkiewicz et al., 2018). Despite the rapidly changing cannabis regulatory landscape, to our knowledge, no studies have utilized GSV to identify or validate cannabis retailers across geographic areas or over time. Therefore, the current study aimed to evaluate the utility and feasibility of using GSV virtual audit methods to systematically and retrospectively validate cannabis retailer locations extracted from online sources across multiple states in the US, addressing a critical methodological gap in enhancing precision and reproducibility of cannabis retailer measures in cannabis research.

## Methods

The visual verification of cannabis retailers was conducted in support of a parent study using National HIV Behavioral Surveillance (NHBS) to examine the impact of cannabis legalization and neighborhood cannabis retailer density on the health of people who inject drugs (PWID) (Redacted for review). The NHBS, created to monitor behavioral risk and protective factors for HIV, surveyed hundreds of PWID in over 20 metropolitan statistical areas (MSAs) with high HIV prevalence every three years (Kanny et al., 2022). The parent study planned to use NHBS data collected during the 2012, 2015, and 2018 PWID cycles. Our study team began collecting cannabis retailer data from websites in 2019. However, datasets capturing the locations cannabis retailers for earlier observation years were not available, necessitating exploration of alternative approaches for developing retrospective databases.

As a result, this study evaluated the feasibility and utility of using GSV to: 1) visually verify cannabis retailers locations extracted from websites and (2) create a historical retailer list. Because of our ultimate goal to develop a retailer list which could be used to create measures of cannabis retailer density for use in the parent study, GSV activities were limited to geographic areas included in NHBS. A total of 13 US jurisdictions (12 states and District of Columbia) included in the NHBS PWID cycles had enacted laws legalizing the sale of cannabis for medical or adult-use any time between 2012 and 2018. Respondents within these 13 jurisdictions lived in 180 unique counties. Due to GSV not being available until 2014, we limited our look back period to 2015, 2016, 2017, and 2018. We visually verified cannabis retailers beginning in 2015 or the year the state first legalized medical cannabis (whichever was later).

### Cannabis Retailer List

In February 2019, we extracted store names, address, and geocoordinates of medical and adult-use cannabis retailers located in the US. Cannabis retailers often opt-in or pay to advertise on websites. In order to provide the most comprehensive view of the market at the time of data collection, data were extracted from 6 websites advertising cannabis retailers: California NORML (canormal.org), kushguide.com, Leafly.com, Weedmaps.com, WeedTraQR.com, and Yelp.com. We removed duplicate observations across websites. Two or more observations were considered duplicates if more than 50% of the characters in the name matched (position and alphabetical character) and the latitude and longitude were within 200 feet (Williams et al., 2023).

**Fig. 1:**
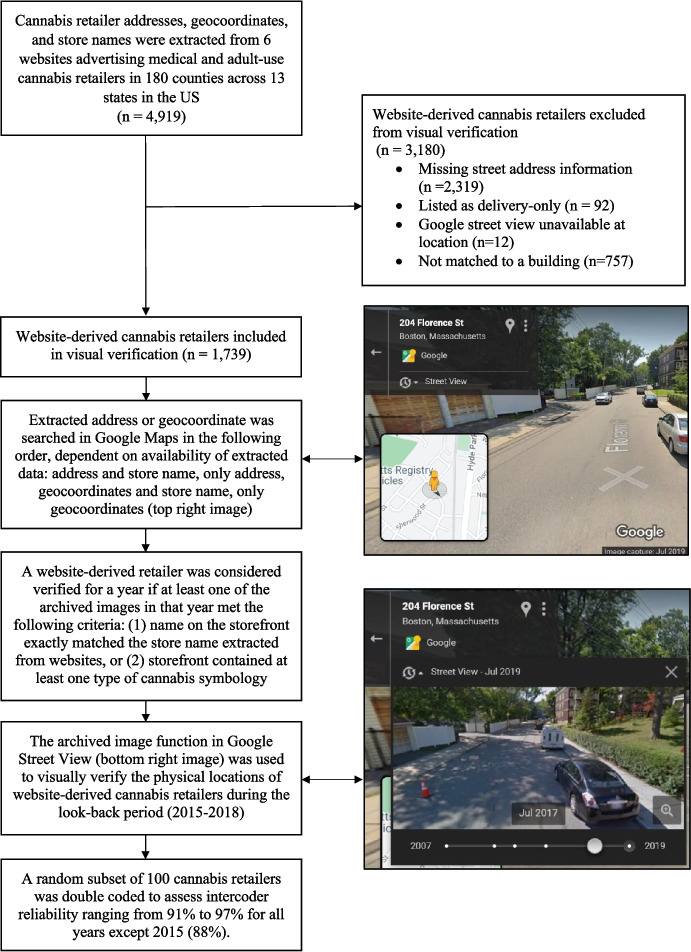
Visual verification of cannabis retailers extracted from websites using Google Street View (GSV) virtual audit

Given the goals of the validation, the dataset was restricted to retailers located in the counties represented in NHBS data. The resulting dataset included 4,919 unique observations in 180 counties across 13 US jurisdictions. Data extracted from websites advertising cannabis retailers (e.g., Weedmaps) require substantial cleaning prior to GSV virtual audit to ensure that the analytic sample is restricted to verifiable brick-and-mortar cannabis retailers. As emphasized in other studies, online listings frequently include delivery-only services, duplicate records, or entries with non-locatable address or not usable data, all of which cannot be reliably assessed using GSV virtual audit methods (Shi et al., 2018; Williams et al., 2023). Prior to visual verification, we followed a multistage data cleaning to restrict lists to likely brick-and-mortar cannabis retailers prior to use. Details are described in full elsewhere (Williams et al., 2023). As part of data cleaning, 2,411 observations were excluded from visual verification because they were missing street address information (*n* = 2,319) or listed as delivery-only retailers, which do not have a brick-and-mortar location (*n* = 92). An additional 849 observations could not be considered for verification because: 1) Google Street View was unavailable at location (*n* = 92) or 2) addresses did not match to a specific building in GSV (e.g., indistinguishable building structures as in the case of a large shopping center or building complexes) (*n* = 757) (Fig. 1). The final analytic dataset included 1,739 observations.

### Google Virtual Audit Training and Oversight

The auditors were graduate students in public health with advanced coursework and applied experience in research methods, data analysis, and spatial data. Prior to data collection, auditors underwent extensive training in Google virtual audits led by a senior member of the study team (Redacted for review). The training included in-depth discussion on all aspects of the virtual audit, including the definition and the appropriate classification of cannabis retailers and documentation of several quality control measures (e.g., storefront symbol descriptions, screenshots of GSV images and image dates). The senior study team member co-created a detailed step-by-step standardized Google virtual audit protocol which guided all procedures, in collaboration with the lead auditor (Redacted for review). The auditors and the senior member met weekly to discuss and troubleshoot the virtual audit throughout the study. During weekly meetings, auditors identified any observations for which they had coding questions. These observations were discussed by the team and resolved within the meeting.

### Historical Google Virtual Audit of Cannabis Retailers using GSV

**Fig. 2:**
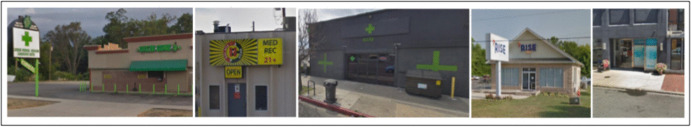
Screen captures of a subsample of visually verified cannabis retailer store fronts. From left to right, store fronts range from clear cannabis symbology to lack of distinct symbology on store front, representing the range of storefront advertising. Image capture from Google 2019

To visually verify cannabis retailers using GSV, each extracted address or geocoordinate in Google Maps was searched in the following order, dependent on availability of data: (1) address and store name, (2) only address, (3) geocoordinates and store name, and (4) only geocoordinates. Website-derived retailers were visually verified using the archived image function in GSV. A website-derived retailer was considered a verified cannabis retailer for a given year if at least one of the archived images in that year met the following criteria: (1) name on the storefront exactly matched the store name extracted from websites, or (2) storefront contained at least one type of cannabis symbology (i.e., cannabis leaf, green or white cross, or cannabis- related terminology). If a retailer was not visually verified in the most recent year available, all available archived images were reviewed to verify whether the retailer was present at any point during the look-back period. For cannabis retailers that were visually verified, auditors also documented the type of cannabis symbology present on the storefront (i.e., cannabis leaf, green or white cross, or cannabis-related terminology). Screen captures of a random subsample (*n* = 524) of verified retailers’ most recent storefronts were saved to capture the variation in cannabis retailer storefronts (Fig. 2).

Visual verification was conducted between May 2019 and February 2020. To assess intercoder reliability, which is a measure of coding consistency between auditors, we selected a random subset of 100 cannabis retailers for double coding in August 2019 (O’Connor & Joffe, 2020). Because a given retailer was assessed across multiple years, this resulted in the double-coding of 382 unique observations. Intercoder reliability was high, ranging from 91%−97% for all years, except for 2015 when it was 88%. Using criteria recommended by Landis and Koch, (Landis & Koch, 1977), ICR values ranging from 0.81 to 1.00 are considered “almost perfect”.

### Analyses

We examined the proportions of retailers visually verified out of the total website-derived cannabis retailers, by year and state, using descriptive statistics. All states legalizing the sale of cannabis for adult use first legalized the sale of cannabis for medical use. The time-period in which a state first legalized the sale of cannabis for medical use has important implications not only for how established the market may be, but also processes governing retailer licensing (and by proxy, timelines and opportunities for market growth following legalization). Pacula and Smart (Pacula & Smart, 2017) grouped state medical cannabis laws into three policy periods, each marked by key political shifts resulting in increased regulatory oversight: the ballot era (1996–2000), the early legislative era (2000–2009) and the late legislative era (2009–2017). Given the heterogeneity in the timing and enactment of cannabis legalization across states, we examined the distribution of verified retailers within and between legislative periods. States were categorized into one of the three legislative periods based on the first effective year of the medical cannabis law using the Prescription Drug Abuse Policy System Medical Marijuana Law Database (Prescription Drug Abuse Policy System, 2024a, 2024b). The Chi-Square test of homogeneity (or Fisher’s Exact test if any expected cell count was below 5) was used to compare proportions of verified retailers within the same legislative period and across the three legislative periods. For comparisons across legislative periods, state verification frequency counts were aggregated within each legislative period. The a priori *P*-value for statistical significance was < 0.05.

## Results

A total of 1,739 website-derived cannabis retailers were eligible for visual verification. Seventy-four percent (*n* = 1,281) were visually verified in at least one year between 2015 and 2018. We observed greater visual verification in more recent years, both within and across states (Table 1). In 2018, visual verification ranged from 41–89%. Visual verification was 74% or greater in all but two states (California [41%], Florida [57%]). We caution interpretation of the low percent verification in Florida due to a small sample size (*n* = 7). Visual verification degraded back in time for most states: in 2017 visual verification ranged from 9–83% and in 2015 it ranged from 0–54%. In California, the state with the greatest number of extracted retailers (*n* = 757), the percentage of website-derived retailers being verified as cannabis retailers was consistently lower than other states sampled, with verification ranging from 24% in 2015 to 41% in 2018.

**Table 1:** Google street view (GSV) visual verification of retailers extracted from websites, by state and by year, 2015–2018 (*n* = 1,739)

Total website-derived cannabis retailers (*n* = 1,739)	Verified cannabis retailer^2^ % (*n*)	Verified as not a cannabis retailer^3^% (*n*)	Missing archived images^4^ % (*n*)
California (*n* = 757)			
2018	41% (310)	49% (369)	10% (78)
2017	37% (283)	59% (445)	4% (29)
2016	26% (199)	66% (500)	8% (58)
2015	24% (178)	73% (553)	3% (26)
Colorado (*n* = 338)			
2018	81% (274)	7% (24)	12% (40)
2017	83% (282)	11% (36)	6% (20)
2016	59% (201)	14% (46)	27% (91)
2015	54% (182)	19% (64)	27% (92)
District of Columbia (*n* = 4)			
2018	75% (3)	25% (1)	0% (0)
2017	75% (3)	25% (1)	0% (0)
2016	75% (3)	25% (1)	0% (0)
2015	25% (1)	25% (1)	50% (2)
Florida (*n* = 7)			
2018	57% (4)	29% (2)	14% (1)
2017	29% (2)	71% (5)	0% (0)
Illinois (*n* = 36)			
2018	86% (31)	11% (4)	3% (1)
2017	67% (24)	22% (8)	11% (4)
2016	36% (13)	33% (12)	31% (11)
2015	8% (3)	50% (18)	42% (15)
Maryland (*n* = 35)			
2018	89% (31)	11% (4)	0% (0)
2017	11% (4)	71% (25)	17% (6)
2016	3% (1)	57% (20)	40% (14)
2015	0% (0)	54% (19)	46% (16)
Massachusetts (*n* = 20)			
2018	85% (17)	10% (2)	5% (1)
2017	55% (11)	30% (6)	15% (3)
2016	25% (5)	45% (9)	30% (6)
2015	0% (0)	20% (4)	80% (16)
Michigan (*n* = 69)			
2018	74% (51)	26% (18)	0% (0)
2017	49% (34)	41% (28)	10% (7)
2016	29% (20)	39% (27)	32% (22)
2015	20% (14)	46% (32)	33% (23)
New Jersey (*n* = 5)			
2018	80% (4)	20% (1)	0% (0)
2017	60% (3)	20% (1)	20% (1)
2016	40% (2)	40% (2)	20% (1)
2015	40% (2)	20% (1)	40% (2)
New York (*n* = 12)			
2018	83% (10)	17% (2)	0% (0)
2017	42% (5)	42% (5)	17% (2)
2016	33% (4)	50% (6)	17% (2)
2015	0% (0)	33% (4)	67% (8)
Oregon (*n* = 266)			
2018	84% (224)	10% (27)	6% (15)
2017	78% (208)	17% (45)	5% (13)
2016	67% (179)	29% (76)	4% (11)
2015	38% (100)	34% (90)	29% (76)
Pennsylvania (*n* = 11)			
2018	82% (9)	18% (2)	0% (0)
2017	9% (1)	91% (10)	0% (0)
2016	0% (0)	73% (8)	27% (3)
Washington (*n* = 179)			
2018	75% (135)	23% (41)	2% (3)
2017	69% (124)	26% (46)	5% (9)
2016	45% (81)	28% (51)	26% (47)
2015	29% (52)	37% (67)	34% (60)

^1^ Results shown only for years in which a state had active medical or adult use cannabis laws

^2^ A website-derived retailer was considered verified for a year if at least one of the archived images in that year met the following criteria: (1) name on the storefront exactly matched the store name extracted from websites, or (2) storefront contained at least one type of cannabis symbology (i.e., cannabis leaf, green/white cross, or cannabis-related terminology)

^3^ Website-derived retailer was considered not verified as a cannabis retailer for the year when there was no visibly matched store name or cannabis symbology present on the storefront in that year

^4^ Website-derived retailer was unable to be visually verified for the year due to a lack of archived images in Google Street View but was visually verified in at least one year during the look-back period

^5^ Website-derived retailer was unable to be visually verified for the year due to a lack of archived images in Google Street View but was visually verified in at least one year during the look-back period

Visual verification in 2018 varied across three legislative periods examined (Table 2). When comparing the proportion of visually verified retailers *among states in a given legislative period*, we found proportions varied within the ballot era (*p* < 0.001), but not in the early legislative period (*p* = 0.236) and late legislative periods (*p* = 0.585). States categorized into the early and late legislative period tended to have fewer total retailers (with the exception of Colorado) but greater visual verification, as compared to the ballot era. Proportions also varied across the three periods (*p* < 0.001). In comparisons *between periods*, proportions varied when comparing the ballot era to early legislative period (*p* < 0.001) and ballot era to late legislative period (*p* < 0.001). In contrast, the proportion of verified retailers did not vary when comparing the early legislative period to late legislative period (*p* = 0.379).

**Table 2:** Google street view (GSV) visual verification results by legislative period in 2018

Total website-derived cannabis retailers by legislative period (*n* = 1,739)	Year of medical cannabis law in effect	Visually verified in 2018 % (*n*)	Verified as not a cannabis retailer or missing archived images in 2018^1^ % (*n*)	*p-*value^2^	
				Within period comparison^3^	Across period comparison^4^
Ballot Era (1996–1999) (*n* = 1,202)					Ballot era vs. Early legislative period: *p* < 0.001 Ballot era vs. Late legislative period: *p* < 0.001 Early legislative period vs. Late legislative period: *p* = 0.379
California (*n* = 757)	1996	41% (310)	59% (447)	< 0.001	
Oregon (*n* = 266)	1998	84% (224)	16% (42)		
Washington (*n* = 179)	1998	75% (135)	25% (44)		
Early Legislative Period (2000–2008) (*n* = 407)					
Colorado (*n* = 338)	2000	81% (274)	19% (64)	0.236	
Michigan (*n* = 69)	2008	74% (51)	26% (18)		
Late Legislative Period (2009 or later) (*n* = 123)					
District of Columbia (*n* = 4)	2010	75% (3)	25% (1)	0.585	
New Jersey (*n* = 5)	2010	80% (4)	20% (1)		
Maryland (*n* = 35)	2013	89% (31)	11% (4)		
Massachusetts (*n* = 20)	2013	85% (17)	15% (3)		
Illinois (*n* = 36)	2014	86% (31)	14% (5)		
New York (*n* = 12)	2014	83% (10)	17% (2)		
Pennsylvania (*n* = 11)	2016	82% (9)	18% (2)		

^1^Includes retailers visually verified as not a cannabis retailer and missing archived images in 2018

^2^ *P*-value based on Chi-Square test of homogeneity with Fisher’s Exact used for comparisons with expected cell counts below 5

^3^ This comparison included states *within* a specified legislative period. For example, the within Ballot Era comparison included California, Oregon and Washington

^4^ For comparisons across legislative periods, state verification frequency counts were aggregated within each legislative period (Ballot: 56% [669/1,202]; Early Legislative Era: 80% [325/407]; Late Legislative Era: 85% [105/123])

Common storefront cannabis-related symbology was identified during visual verification. Among the 1,281 retailers visually verified in at least one year, types of cannabis-related symbology included the image of a green or white cross (39%, *n* = 498), the image of a cannabis leaf (14%, *n* = 175), and cannabis-related terminology (12%, *n* = 150). The remaining retailers had no storefront cannabis-related symbology present, but included store name (36%, *n* = 458).

## Discussion and Conclusions

This study utilized GSV virtual audit methods to visually verify the physical locations of website-derived cannabis retailers across 180 counties in 13 jurisdictions in 2015, 2016, 2017, and 2018. We found that for most states sampled (with the exception of California), visual verification was high for the time-period contemporaneous to data collection, but decreased further back in time. Collectively, our findings suggest that GSV virtual audit methods may be appropriate for visually verifying retailers contemporaneously in many US jurisdictions legalizing cannabis sale, but are not appropriate for reconstructing historic retailer lists.

Visual verification was 74% or greater in all but two states in 2018. This finding is comparable with a study verifying cannabis retailer locations by telephone across 26 states, which found that 79% of website-derived cannabis retailers had correct addresses listed on websites (Williams et al., 2023). When examining visual verification within and between legislative periods, we found visual verification varied within the ballot era and between the ballot era and both the late and early legislative periods. The variation observed within the ballot period and between legislative periods when compared to the ballot era was likely driven by California (where only 41% of retailers were verified), as compared to 75% in Washington and 84% in Oregon. In California, the state with the greatest number of extracted retailers, the percent of website-derived retailers verified as cannabis retailers was consistently lower than other states sampled, across all time periods. These results were comparable with past studies using online and telephone verification of website-derived cannabis retailers in California, in which 39% (Cao et al., 2020) to 64% (Pedersen et al., 2018, 2020; Williams et al., 2023) of retailers in California were verified. Retailers in California are regulated by local jurisdictions resulting in a range of local regulations (Freisthler & Gruenewald, 2014; Freisthler et al., 2013; Pacula & Smart, 2017; Thomas & Freisthler, 2017). Shifts in local regulations may promote frequent retailer turnover (Fuller, 2019; Thomas & Freisthler, 2017). Because websites may not remove retailers once closed, this may result in overestimation of retailers in online data sources. Additionally, low verification may be due in part to the existence of a robust unregulated retail market (Herrington, 2019; Thomas & Freisthler, 2017). Unlicensed retailers may be less likely to utilize distinct storefront advertising required for visual verification (Herrington, 2019; Thomas & Freisthler, 2017). Clarity of cannabis symbology on retailer storefronts is a criterion for using Google virtual audit methods to verify the physical locations of retailers (Clarke et al., 2010; Nesoff et al., 2020; Rundle et al., 2011; Rzotkiewicz et al., 2018). Collectively, our findings, placed in context of other work demonstrating low verification of California retailer lists across multiple verification methods (e.g., phone, online) underscores the need for a multimodal, robust approach to validating retailer lists (Cao et al., 2020; Pedersen et al., 2018, 2020; Williams et al., 2023).

Our ability to visually verify the physical locations of retailers decreased the further we went back in time. Reasons for low visual verification varied. Low visual verification in 2017 was driven by small sample size (several states sampled had newly enacted medical laws and small retailer counts). In contrast, low visual verification in 2015 was largely driven by missing archived images. Past studies also identified infrequent historical image capture as a limitation of GSV (Rzotkiewicz et al., 2018). GSV was launched in 2014. Utilizing GSV to verify website-derived retailers may be a more useful tool in more recent years as GSV continues to expand. Of importance, all of the counties included in this study were in urban areas due to the parent study design. Past work has found that GSV may not be appropriate for identifying business establishments in rural settings (Crawford et al., 2019); area urbanicity should be considered when selecting verification methods. In instances where GSV images were available, lower visual verification may have been due in part to rapid turnover (e.g., closure) of retailers or new market growth over time (Amiri et al., 2019; Freisthler et al., 2016; Thomas & Freisthler, 2017). GSV may be a useful tool to track the existence of a single retailer over time (e.g., to identify whether a retailer was open at a given address for multiple years) in instances where images are available, but is not appropriate for reconstructing historic retailer lists more broadly, as it will not identify historic retailers at addresses not included in the source dataset. The feasibility and utility of using alternative approaches, such as machine learning methods, to construct historic lists should be explored (Doiron et al., 2022).

Our study is among the first to verify the physical locations of website-derived cannabis retailers using GSV virtual audit methods across multiple states and years. This study used robust data cleaning methods to remove duplicate or incomplete observations prior to visual verification. Other studies have also emphasized the importance of excluding incomplete cannabis retailer addresses extracted from websites (Shi et al., 2018; Williams et al., 2023). However, several limitations should be noted. Findings may not be generalizable to jurisdictions not included in this study, due to variability in state cannabis markets or area urbanicity. We utilized a single approach to verifying cannabis retailers extracted from websites. This method was closely tied to the availability and quality of archived GSV images. Therefore, cannabis retailers may have been missed due to poor image resolution in GSV. Additionally, variation in laws governing storefront advertising of cannabis products (e.g., limiting storefront signs to only include the name of the business, not allowing depictions of a plant or leaf on storefronts), may have hindered the virtual audit verification process (Leafly, 2022).

In summary, this study used GSV virtual audit methods to systematically and retrospectively validate cannabis retailer locations extracted from online sources across multiple states in the US, addressing a critical methodological gap in enhancing precision and reproducibility of cannabis retailer measures in cannabis research. Cannabis retailer lists obtained from websites require robust data cleaning to remove extraneous observations (e.g., items missing address information). We found that using GSV virtual audit methods may be appropriate for visually verifying retailers contemporaneously, but is not appropriate for reconstructing historic retailer lists. The appropriateness of GSV as a verification tool depends on several factors, including GSV coverage in the geographic area(s) under consideration, urbanicity, and local regulations governing storefront advertising by cannabis retailers. Some states, such as California, which has demonstrated low verification of retailers across multiple validation methods (e.g., phone, online) may require the use of multimodal verification methods. High-quality longitudinal data across multiple states are needed to inform evidence-based policy approaches to cannabis legalization. Future studies should assess the feasibility and validity of using GSV in conjunction with novel approaches, such as machine learning methods, and as well as other approaches previously used for verifying medical and adult-use cannabis retailers (e.g., calling retailers, in-person site visits).

## Data Availability

The datasets used and/or analyzed during the current study are available from the corresponding author on reasonable request.

## Abbreviations

GSV: Google Street View
US: United States
PWID: People who inject drugs
NHBS: National HIV Behavioral Surveillance
